# Genotyping Squamous Cell Lung Carcinoma in Colombia (Geno1.1-CLICaP)

**DOI:** 10.3389/fonc.2020.588932

**Published:** 2020-12-15

**Authors:** Andrés F. Cardona, Alejandro Ruiz-Patiño, Oscar Arrieta, Luisa Ricaurte, Zyanya Lucia Zatarain-Barrón, July Rodriguez, Jenny Avila, Leonardo Rojas, Gonzalo Recondo, Feliciano Barron, Pilar Archila, Carolina Sotelo, Melissa Bravo, Nataly Zamudio, Luis Corrales, Claudio Martín, Christian Rolfo, Lucia Viola, Hernán Carranza, Carlos Vargas, Jorge Otero, Maritza Bermudez, Tatiana Gamez, Luis Eduardo Pino, Rafael Rosell

**Affiliations:** ^1^ Clinical and Translational Oncology Group, Clínica del Country, Bogotá, Colombia; ^2^ Department of Medical Oncology, Foundation for Clinical and Applied Cancer Research – FICMAC, Bogotá, Colombia; ^3^ Molecular Oncology and Biology Systems Research Group (Fox-G), Universidad el Bosque, Bogotá, Colombia; ^4^ Thoracic Oncology Unit, Instituto Nacional de Cancerología (INCan), México City, México; ^5^ Oncology Department, Clínica Colsanitas, Bogotá, Colombia; ^6^ Thoracic Oncology Section, Centro de Educación Médica e Investigaciones Clínicas – CEMIC, Buenos Aires, Argentina; ^7^ Oncology Department, Hospital San Juan de Dios, San José Costa Rica, Costa Rica; ^8^ Medical Oncology Group, Fleming Institute, Buenos Aires, Argentina; ^9^ Marlene and Stewart Greenebaum Comprehensive Cancer Center, University of Maryland School of Medicine, Baltimore, MD, United States; ^10^ Thoracic Oncology Unit, Fundación Neumológica Colombiana, Bogotá, Colombia; ^11^ Department of Medical Oncology, Fundación Santa Fé de Bogotá, Bogotá, Colombia; ^12^ Department of Medical Oncology, Catalan Institute of Oncology, Barcelona, Spain

**Keywords:** squamous cell carcinoma, lung cancer, genotype, PD-L1, Latin America, therapeutic target

## Abstract

**Background:**

Lung cancer is a public health problem, and squamous cell carcinoma (SCC) is the second most prevalent subtype of this neoplasm. Compared to other subtypes, including adenocarcinoma, SCC is less well understood in terms of molecular pathogenesis, limiting therapeutic options among targeted agents approved for other disease subgroups. In this study, we sought to characterize the SCC genomic profile using a validated Next Generation Sequencing (NGS) platform.

**Methods:**

The comprehensive NGS assay (*TruSight Tumor 170*) was used in order to target the full coding regions of 170 cancer-related genes on SCC samples. PD-L1 expression in tumor cells (TCs) was assessed using clone 22C3 (Dako). Clinical outcomes were correlated with molecular profile, including progression free survival (PFS), overall response rate (ORR), and overall survival (OS).

**Results:**

A total of 26 samples were included, median age was 67 years (r, 33–83) and 53.8% were men. Tobacco consumption was identified in all subjects (mean 34-year package). For first-line treatment 80.8% of patients received cisplatin or carboplatin plus gemcitabine. In terms of molecular profile, we identified a high prevalence of inactivating mutations in TP53 (61.5%), PIK3CA (34.6%), MLL2 (34.6%), KEAP1 (38.4%), and NOTCH1 (26.9%). PD-L1 expression ranged from negative, 1, 2–49, and ≥50% in 23.1, 38.5, 26.9, and 11.5%, respectively. Interestingly, the genetic alterations did not have an effect in PFS, OS or ORR in this study. However, PDL1 expression was higher among those who had mutations in TP53 (p = 0.037) and greater expression of PDL1 was related to PIK3CA alterations (p = 0.05).

**Conclusions:**

The genomic profile of SCC encompasses important genes including TP53, PIK3CA and KEAP1. TP53 mutations could be associated with PDL1 expression, generating hypothesis regarding specific treatment options.

## Highlights

No targeted therapeutics are approved for treatment of squamous cell lung cancer (SCC), largely because of a scant understanding of its molecular pathogenesis. We evaluated 26 cases of Colombian patients with SCC using next-generation sequencing and PD-L1 immunohistochemistry in tumor samples. We identified a relatively high prevalence of inactivating mutations in TP53, PIK3CA, MLL2, KEAP1, and NOTCH1 as previously described in the literature. Lower PD-L1 expression was observed in samples with alterations in TP53 and PIK3CA.

## Introduction

The incidence of squamous cell carcinoma of the lung (SCC) has steadily fallen while the incidence of lung adenocarcinoma (LUAD) has risen over the last few decades. This is due to decreased smoking rates and changes in cigarette filtering and composition (1). Nevertheless, lung SCC remains a common malignancy overall, accounting for approximately 85,000 new cases in the United States each year and over 400,000 worldwide (2). The great majority of patients with lung SCC are current or former heavy smokers, in contrast to LUAD, in which case a growing proportion of patients are never-smokers or former light smokers (3). The situation in Latin-American countries is not different, approximately 75,000 new cases of lung cancer are diagnosed yearly. Lung cancer constitutes the leading cause of cancer deaths for both sexes, and accounts for 12% of all cancer-related deaths (4). In regards to histologic type, LUAD has the highest incidence rate in most Latin-American countries, but SCC incidence rates are similar to those reported for LUAD (Uruguay, Costa Rica, Pasto in Colombia, Aracaju and Cuiaba in Brazil, Mendoza in Argentina) and even higher in some specific regions (Antofagasta region in Chile, Manizales in Colombia for males, and Villa Clara in Cuba) (5). Interestingly, in the region of Central and South America there is a strong association between prevalence of current smoking and lower income levels, therefore it is likely that tobacco-related diseases, including SCC, disproportionately affect vulnerable population subgroups (6).

During recent decades there has been great interest in understanding the genomic landscape of the different histologic subtypes of lung cancer and its therapeutic potential. In LUAD, patterns of specific oncogenic mutations vary widely among different regional subgroups. *EGFR* mutations present a higher prevalence in populations from East Asia and Latin-America while *KRAS* mutations are present in a lower rate compared with Caucasians (7–10). Because of this, LUAD therapeutics have become personalized and clinical trials are now designed accordingly in order to supply this emerging need. In contrast, lung SCC is an area of unmet need in lung cancer research. It is known that the complex genomic landscape and high overall mutational burden present in lung SCC is directly related to the protagonist role of tobacco pertaining to carcinogenesis. In fact, lung SCC appears to have a somatic mutation rate similar to small cell lung cancer (SCLC) and other smoking related malignancies, while LUAD (non-smoker related cancer) harbors a much lower rate of somatic mutations (11, 12). Moreover, lung SCC genomic alterations are very similar to Human Papilloma Virus (HPV) negative head and neck cancers (13). To date, different genomic subsets of lung SCC have been studied and identified, some with important therapeutic implications for a growing number of developing targeted agents (14). Previous studies identified amplifications of *p63, PI3KCA, PDGFRA, SOX2*, or *FGFR1*, mutations in *TP53, EGFRvIII, PI3KCA, NRF2, PTEN*, and *DDR2* using single nucleotide polymorphisms arrays (SNP), focused DNA sequencing and gene expression profiling (11, 15).

In 2012, The Cancer Genome Network Atlas (TCGA) profiled 178 SCC and provided a comprehensive landscape of the genomic and epigenomic alterations of this entity. They identified that SCC is characterized by a mean of 360 exon mutations, 165 genomic rearrangements, and 323 segments of copy number alteration per tumor. Additionally, they found statistically recurrent mutations in 11 genes (*TP53, CDKN2A, PTEN, PIK3CA, KEAP1, MLL2, HLA-A, NRF2, NOTCH1, RB1*, and *HLA-A*) and copy number alterations of chromosomal segments that contained *SOX2, PDGFRA* and/or *KIT, EGFR, FGFR1* and/or *WHSC1L1, CCND1, CDKN2A, NFE2L2, MYC, CDK6, MDM2, BCL2L1, EYS, FOXP1, PTEN*, and *NF1 TP53* mutations were present in 81% of the cases and almost all displayed selective amplification of chromosome 3q. They also reported frequent modifications in genes enrolled in squamous differentiation (*SOX2/TP63/NOTCH1*, 44%), apoptotic signaling (*PI3K/AKT*, 69%), cell cycle control (*CDKN2A/RB1*, 72%), and response to oxidative stress (*NFE2L2/KEAP1/CUL3*, 34%). Another important finding from this study is that even though alterations in *EGFR* and *KRAS* are uncommon, approximately 30% of the cases present mutations and amplifications in the *PI3K/AKT* pathway and in tyrosine-kinase receptors *EGFR, FGFR*, and *BRAF* (16). Afterwards, a study carried out in 2014 performed whole DNA exome sequencing in 104 Korean patients with lung SCC. They encountered a mean of 261 somatic exon mutations per tumor and found 7 statistically significant gene alterations (*TP53, PTEN, NFE2L2, KEAP1, MLL2*, and *PIK3CA*). When compared with the TCGA study, the genomic landscape of lung SCC demonstrated a similar spectrum of alterations between the Korean and the North American populations, contrary to the case of LUAD in which demographic variation is prominent (17).

After a decade of comprehensive genomic evaluation in NSCLC, SCC specific prognostic gene signatures and prognostic predictive biomarkers are emerging. Nevertheless, the genomic landscape of lung SCC among the Hispanic population is still unknown. This study probed 170 key lung SCC cancer genes retrieved from previous reports through next generation sequencing (NSG) in 26 tumor samples from Colombian patients. Additionally, PD-L1 baseline expression was evaluated and correlated with the genomic landscape.

## Methods

Twenty-six Colombian patients with histological diagnosis of lung SCC (positivity for TTF1 and p40) and locally advanced or advanced disease (AJCC stages IIIA/IIIB/IV) treated in Bogota, Colombia between January 2010 and June 2016 were selected for the study. These patients had tissue availability and a complete follow-up. An Institutional Review Board and Privacy Board waiver was obtained to facilitate retrospective collection of clinical-pathologic and molecular data (Geno1.1-CLICaP Platform - Registration No. 2011/048, Clínica del Country, Bogotá, Colombia). Clinical data collected were age, gender, Eastern Cooperative Oncology Group (ECOG) performance status, TNM and sites of metastases. All samples were histologically reviewed, evaluated for PD-L1 expression with immunohistochemistry (IHC) and analyzed by Next Generation Sequencing (NGS). None of the cases presented a mixed histology component.

### Tissue Selection, Management, and Evaluation

Tumor morphology and all IHC stained sections were evaluated and scored by an expert pathologist (PA) and discrepancies were resolved by a complementary external evaluation (control). The standard immunohistochemical panel included at least TTF-1 and either CK5, CK7 or p40. The suitability of material for mutational analysis was assessed based on hematoxylin and eosin (H&E) stains of FFPE tissue blocks and/or cytology specimens (if available). A representative area with high frequency of malignant cells was identified, from which sections for mutational analysis were taken followed by new H&E sections to ensure that a representative material had been taken. An estimate of tumor cell content was made by a diagnostic pathologist, with a requirement of ≥10% for the mutational analysis. In addition to FFPE tissue blocks, tissue material for mutation analysis could also originate from cytology slides, or sections from centrifuged and paraffin embedded cytology material (cell blocks). Sections were stored at -20°C until nucleic acid extraction.

In case of preparation of cell lysate from cytology slides, a representative tumor cell rich area of a cytology slide was identified, the slide was scanned (to enable future clinical review), and the glass cover slip was removed using xylene followed by a rehydration step in ethanol. Thereafter, the cells were lysed using 180 ul ATL Buffer from Qiagen (Qiagen, Hilden, Germany). DNA was extracted from the lysate within 24 h and stored at -20°C.

### PD-L1 Expression

IHC analysis was carried out in previously deparaffinized tissue sections. Rehydration and posterior antigen retrieval were performed using XS Tris Buffered Saline with Tween 20 and boiled for 20 min. Rabbit monoclonal primary PD-L1 antibody (Monoclonal Mouse Anti-Human PD-L1 Clone 22C3, Agilent Technologies, Santa Clara, California, US) was processed using 4 mm-thick FFPE tissue sections on a EnVision FLEX visualization system on Autostainer Link 48 (Agilent Technologies, Santa Clara, California, US) with standard antigen retrieval methods. The Signal Stain DAB substrate kit (#8959) was used according to the manufacturer´s instructions. Human placenta was used as positive control. PD-L1 protein expression was determined using Tumor Proportion Score (TPS), which is the percentage of viable tumor cells showing partial or complete membrane staining at any intensity. Tumors with ≥1% of tumor cells stained either in membrane or cytoplasm will be considered positive for PD-L1. The grading system of PD-L1 expression was: 0 (negative), 1–49%, (weak to moderate expression), and >50 (strong expression).

### DNA and RNA Extraction

DNA and RNA for NGS-based mutation analysis were extracted using the Qiagen AllPrep kit for FFPE tissue and automated on the QIAcube instrument (Qiagen, Hilden, Germany). The protocol was modified with an extended proteinase K digestion (overnight) for the DNA extraction to obtain higher DNA yields. DNA from cytology slides was extracted using the QiaAmp DNA Micro kit (Qiagen, Hilden, Germany). RNA was not extracted from cytology specimens. Following extraction, DNA samples were quantified using Qubit and RNA samples were quantified using a BioAnalyzer. Then, for quality assessment DNA samples were analyzed by qPCR using the Illumina FFPE QC Kit (WG-321-1001) along with a control cell line sample with a known input mass.

### NGS-Based Mutational Analysis

NGS-based mutation analysis was performed using the Illumina TruSight Tumor 170 (TST-170; Illumina, Inc., San Diego, California, US), an NGS assay designed to cover 170 genes associated with solid tumors. TST-170 is an enrichment-based targeted panel that simultaneously analyzes DNA and RNA, identifying somatic mutations as low as 5% mutant allele frequency with ≥250× minimum coverage. The panel includes 55 genes for fusions and splice variants, 148 SNVs and indels, and 59 amplifications (**Supplementary Table 1**). After quantitation and quality assessment, samples that met the minimum input threshold (3.3 ng/µl for DNA, 4.7 ng/µl for RNA), were processed through the TST-170 Kit assay (NextSeq v2.5 Reagents - Cat. No. 20028821). DNA samples were sheared for library preparation and RNA samples were converted to cDNA. Both sample types were then run in parallel through library preparation followed by a hybrid capture enrichment targeting 170 key cancer genes (https://www.illumina.com/content/dam/illumina-marketing/documents/products/datasheets/trusight-tumor-170-data-sheet-1170-2016-017.pdf).

Samples were evaluated for performance based on quality control (QC) metrics established during the development of the TST-170 assay. Subsequently, both sample types were run in parallel through library preparation followed by a hybrid capture enrichment. The TMB (Tumor Mutation Burden) determination of the TST-170 was based on variant calls of the Illumina pipeline (local BaseSpace application) and only considered non‐synonymous variants filtered against population frequency (ExAC and 1000G, only variants <0.0001% MAF). All variant calls were manually curated blinded to the TMB calls to exclude false positive and negative variants before TMB calculation. Variant interpretation was based on recommendations from the Association for Molecular Pathology (AMP), American Society of Clinical Oncology (ASCO), and College of American Pathologists (CAP). The predictive value of the genes with variants evaluated was only for single gene changes. If applicable, actionable genetic alterations were stratified into one of four levels based on OncoKB website (http://www.OncoKB.org). Tier 1 variant included level 1 and level 2 genetic alterations that are Food and Drug Administration–approved biomarkers and standard of care. Tier 2 variant included alterations with compelling clinical or preclinical evidence to drug response.

### Statistical Analysis

For descriptive purposes, continuous variables were summarized as arithmetic means, medians, and standard deviations. Categorical variables were reported as proportions with 95% confidence intervals (95% CIs). Inferential comparisons were performed using Student’s t test. χ^2^ or Fisher’s exact test were used to assess the significance among categorical variables. The time-to-event variables obtained from the Kaplan-Meier method were determined by log-rank tests. To test the association between mRNA expression (continuous variables) and clinic-pathologic features (dichotomous variables), the Kruskal-Wallis and Mann-Whitney U tests were used. Statistical significance was determined as *p ≤*0.05 using a two-sided test. All the statistical analyses were performed using SPSS version 19.0 (SPSS, Inc., Chicago, IL, US).

## Results

### Patient Characteristics

Patient demographic and clinical characteristics are displayed on **Table 1**.** **A total of 26 patients met criteria and had available tumor samples and were therefore included in the analysis. The age of the patients ranged between 33 and 83 years, with a median of 67 years. Eighteen (18/26; 69.2%) patients were former smokers and eight (8/26; 30.8%) were current smokers. The median cigarette exposure was 34.4 packs-year. 21 patients presented with advanced disease (AJCC stage IV) and received cisplatin or carboplatin plus gemcitabine as first line therapy. The most common primary site of metastasis besides lungs and pleura (65.4%) was bone (11.5%). The median follow-up for the cohort was 15.8 months (95%CI 11.2–19.4) and 27% of patients were alive at the time of last follow-up. The NGS-tested specimens included 17 FFPE sections taken from the primary (65.4%), followed by visceral and lymph node metastases in 6 cases (23%). Only in one case the sample was obtained from cytology plus a cell block from pleural effusion. The median turnaround time (TAT) for the entire molecular process (from clinical referral, pathological evaluation, and molecular analysis, to the final report) was 17 calendar days (mean 15 ± 4 calendar days).

**Table 1:** Demographic characteristics of Colombian patients with lung SCC.

Variable	Colombian cohort N(%)
**Age (median, range)**	67 (33–83)
>65 years	14 (53.8)
<65 years	12 (46.2)
**Gender**	
Male	14 (53.8)
Female	12 (46.2)
**Tobacco exposure**	
Pack year	34.4 (SD +/-16.8)
Former smoker	18 (69.2)
Current smoker	8 (30.8)
**T**	
1	5 (19.2)
2	13 (50.0)
3	4 (15.4)
4	4 (15.4)
**N**	
0	6 (23.1)
1	6 (23.1)
2	7 (26.9)
3	7 (26.9)
**M**	
0	5 (19.2)
1	21 (80.8)
**Initial AJCC stage**	
IIIa	1 (3.8)
IIIb	4 (15.4)
IV	21 (80.8)
**Primary site of metastases**	
Lung/Pleural	17 (65.4)
Bone	3 (11.5)
Suprarenal	1 (3.8)
Brain	2 (7.7)
Liver	3 (11.5)

### Molecular Profile

**Figure 1:**
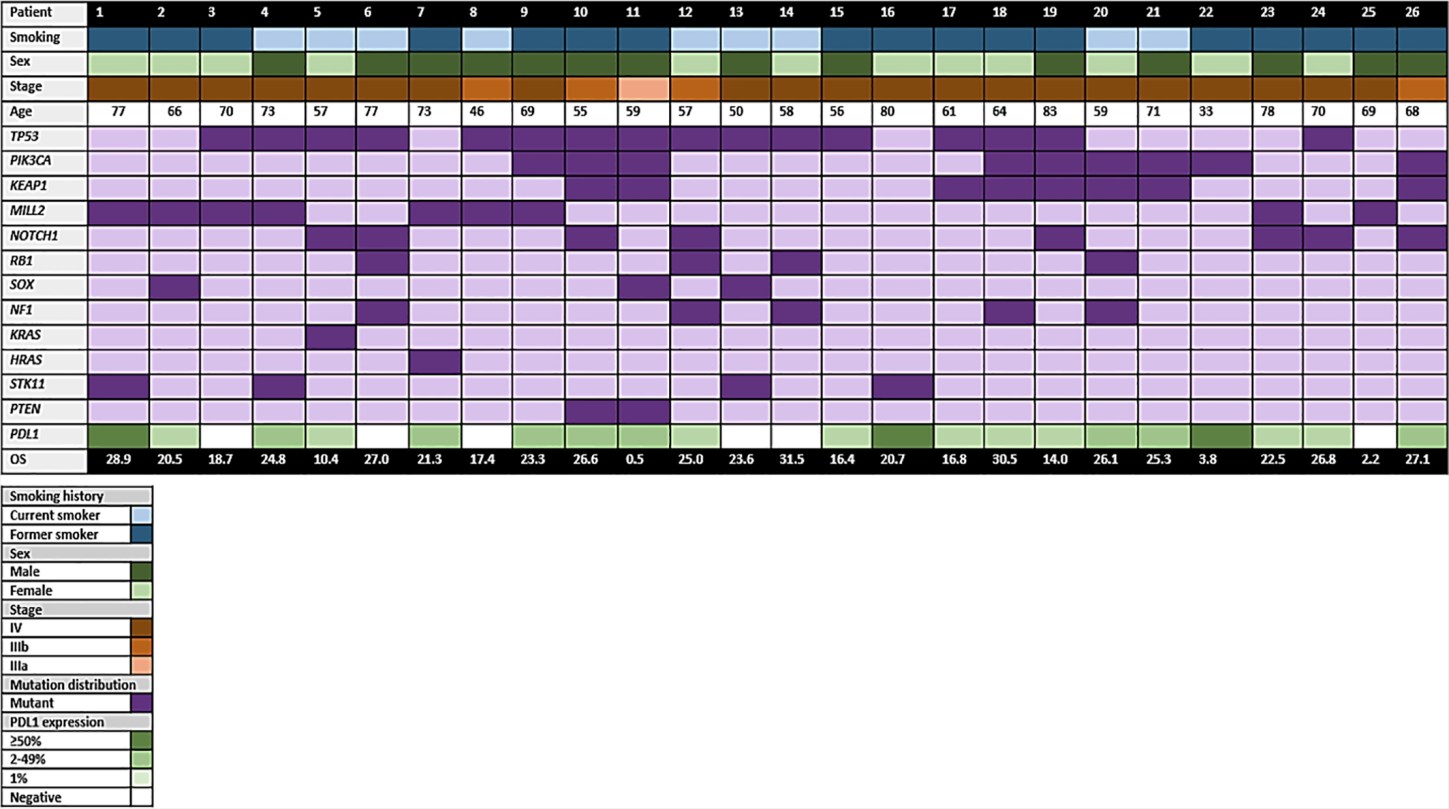
Diagram highlighting the main characteristics of the patients included in the study.

DNA and RNA were successfully extracted in all cases. 26 DNA libraries were analyzable with DNA concentrations between 3.6–214.4 ng/μl (median 75 ng/μl) and tumor cell content between 20 and 60%. Overall, 98% of the targeted sequences had at least 30×target base coverage across the samples. Mutational analysis detected 451 DNA sequence changes; these included 187 missense mutations, 55 truncating mutations, and 9 in-frame deletions. The most significantly amplified regions were 3q26, an amplicon including SOX2; 3q26 (PIK3CA); 8p11 (FGFR1, NSD3); 7p11 (EGFR); 11q13 (CCDN1); and 4q12 (KDR, KIT, PDGFRA). Deletion peaks tended to be broader and more included 2q37, 4q35 (CASP3), 9p21 (CDKN2A), and 10q23 (PTEN). We also found a relatively high prevalence of inactivating mutations in TP53 (61.5%), MILL2 (34.6%), PIK3CA (34.6%), KEAP1 (38.4%), NOTCH1 (26.9%), and NF1 (19.2%). Other genetic alterations identified were: RB1, SOX, KRAS, NRAS, STK11, and PTEN. **Figure 1** shows the relationship between the main clinical characteristics and the molecular profile of each included patient.

The TMB determination of the TST-170 data was based on variant calls of the Illumina pipeline (*local BaseSpace application*) and only considered non‐synonymous variants filtered against population frequency (ExAC and 1000G, only variants <0.0001% MAF). All variant calls were manually curated blinded to the TMB calls to exclude false positive and negative variants before TMB calculation. The range of TMB as detected by panel sequencing for the cohort was between 4 and 34 Mut/Mbp (mean 15.6+/-9). In addition, 65% of patients with a TMB ≥10 Mut/Mbp had a history of tobacco exposure greater than 10 years/pack (*p* = 0.037); however, a linear relationship was not identified between TMB and PD-L1 expression level [most of the patients with TMB ≥10 Mut/Mbp (n = 13/93%) had a PD-L1 <50%; *p* = 0.63]. The presence of a TMB ≥10 Mut/Mbp was not related with an incremental number of mutations in TP53 (*p* = 0.46), in PI3K (p = 0.085), in KEAP1 (*p* = 0.15) or in MLL2 (*p* = 0.13). Similarly, a higher TMB (≥ 10 Mut/Mbp) was also not associated with older age (*p* = 0.20) or with the number of metastatic sites (*p* = 0.72) or the site of location of metastases including brain (p = 0.59). **Supplementary Figure 1** discriminates the type of alteration for the genes with the highest prevalence in the cohort of Colombian patients with lung SCC.

### PD-L1 Expression

**Figure 2:**
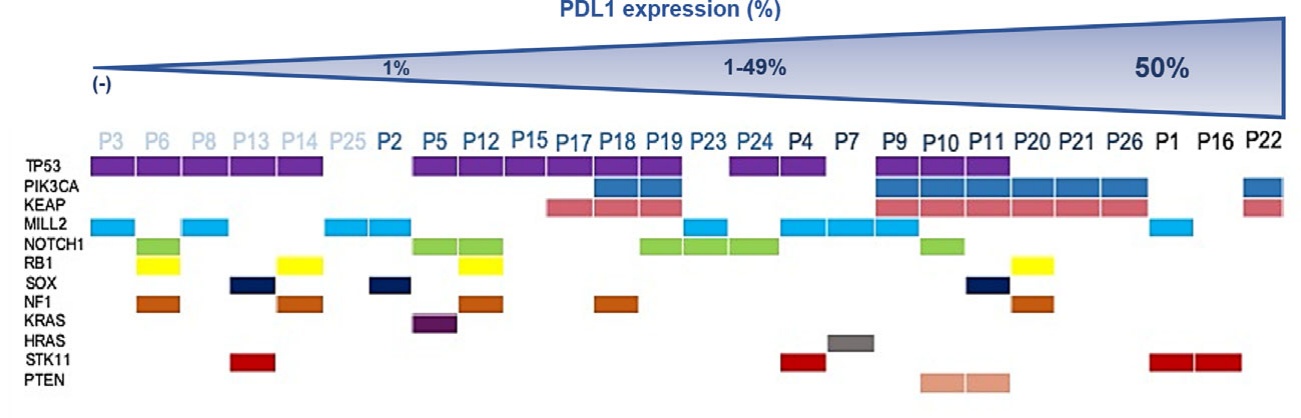
Distribution of PDL1 expression among main genomic alterations found in Colombian patients with lung SCC.

PD-L1 expression was ≥50, 1–49 and negative in 11.5, 34.6, and 23.1%, respectively. PD-L1 was positive in 20 patients (n = 20/77%) with history of tobacco exposure (15 former smokers and 5 current smokers), being higher in the group with a consumption rate of 20 years/package (n = 15/75%). PD-L1 positivity was greater in patients with missense mutations in TP53 and PI3K (*p* = 0.04), as opposed to those who had mutations in MLL2 (*p* = 0.69). Interestingly, 4 patients presented with coexisting mutations of TP53, PIK3CA and KCAP1 had PDL1 levels ≥50%. **Figure 2** shows the distribution of PDL1 expression among the main genomic alterations found in Colombian patients with lung SCC.

### Treatment and Survival According to Molecular Characteristics

**Figure 3:**
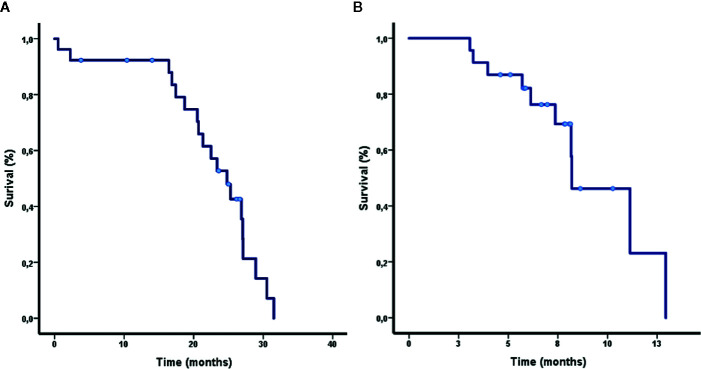
**(A, B)** Overall survival and progression free survival for first line treatment in patients included in the study.

Eighty percent of patients received cisplatin or carboplatin plus gemcitabine as first line treatment with an overall response rate (ORR) of 61.5% (complete response 1/3.8%, partial response 15/57%, stable disease 8/30.8%, and progressive disease 2/7.7%) and a median progression free survival (PFS) of 8.2 months [95% Confidence Intervals (CI): 5.6–10.7]. Most patients received docetaxel as second line (n = 22/84.6%), in 3 cases the treatment was with gemcitabine/vinorelbine (11.5%) and 1 subject was lost to follow-up. The ORR and PFS for the second line were 38.5% and 5.1 months (95% CI 4.6–8.1), respectively. Of the 9 patients who received Nivolumab as third line, 5 achieved a partial response as best outcome (4 cases with TMB ≥10 Mut/Mbp/PD-L1 1-49% and 1 case with TMB ≥10 Mut/Mbp/PD-L1 negative). The median PFS for nivolumab-responsive patients with a TMB ≥10 was 8.9 months (95% CI 7.4–10.4). The whole cohort reached an overall survival (OS) of 24.8 months (95% CI 20.8–28.7) (OS and first line PFS are displayed on **Figures 3A, B**, respectively). Most genetic alterations had no effect in terms of OS or ORR, while PI3KCA was associated with a higher first line PFS [HR 6.8, (95% CI 1.5–31); p = 0.012]. PDL1 moderate (1–49%) and strong expression (> 50%) were positively correlated with a prolonged OS when compared to negative PDL1 expression (**Table 2**).

**Table 2:** Overall Survival according to PDL1 expression.

PD-L1 expression level	Overall survival(months)	95%CI	
		Lower Limit	Upper limit
**Negative**	18,7	9,4	27,9
**1-49%**	24,8	21,1	28,4
**>50%**	20,7	20.2	28.1
**Global**	24,8	20,8	28,7

## Discussion

To the best of our knowledge, this is the first study that attempted to genotype SCC among a Colombian population. The patients included in three different cohorts (Korea, North America, and Colombia) show somewhat comparable mean age and current/former smoker status. **Table 3** displays the clinical and molecular characteristics of the population from these cohorts. It is important to note that the presence or absence of demographic heterogeneity in the TCGA study was not mentioned; hence we assume most of the patients are of Caucasian origin. The identification of 12 gene mutations among our cohort and the fact that all of the patients had significant cigarette smoking history is concordant with the complex and densely mutated genomic landscape previously described in lung SCC related to the high mutagenic influence of tobacco (11, 12). The genomic profile seen in the 26 patients with lung SCC is similar to the previously reported in studies carried out in Asian and North American cohorts (11, 19–21). These findings further strengthen the notion that lung SCC is a relative homogeneous malignancy throughout the different demographic groups, in contrast to LUAD. (11, 12) It is important to highlight that in this study, we were unable to identify any significant associations between specific gene variants and baseline characteristics, including the site and number of metastases, though this might be due to a limited sample size.

**Table 3:** Comparison of the clinical and molecular characteristics between lung SCC from the TCGA (16), Korean (18), and Colombian cohorts.

Variable	TCGA^*^(n = 178)	Asians^**^(n = 104)	Colombia(n = 26)
Age (Median)	68	65	67
Males	73.6%	96.2%	53.8%
Male/Female ratio	2.78	25.0	1.16
Stage III/IV	41 (38.2)	20 (19.2)	25 (96.1)
Current/Former smoker	165 (92.6)	99 (95.1)	26 (100.0)
TP53	81%	73%	61%
RB1	7%	15%	15%
MLL2	20%	24%	34%
PTEN	8%	11%	7%
NFE2L2	15%	17%	–
KEAP1	12%	16%	30%
NOTCH	8%	15%	15%
SWI/SNF	–	15%	–
CHD7	–	13%	–
NF1	11%	12%	19%
PIK3CA	16%	9%	34%
WNT	–	5%	–
FBXW7	–	4%	–
CDKN2A	15%	3%	4%
TIE1	–	5%	–
KDM6A	–	5%	–
IBTK	–	3%	–
EGFR	9%	2%	–
RET	–	3%	–
STAT3	–	2%	–
ALK	–	4%	–
FGFR	7%	5%	4%
KRAS	3%	3%	4%
HRAS	3%	–	4%
SOX2	21%	–	11%
STK11	2%	–	15%
CUL3	7%	2%	4%
FOXP1	4%	–	–
ASCL4	3%	–	1%
HLA-A	3%	–	–

^*^Nature. 2012 Sep 27; 489(7417): 519–525. ^**^J Clin Oncol. 2014 Jan 10; 32(2): 121–128.

There are several differences between these studies. First, the TCGA cohort had around 40% of patients with advanced disease; for the Korean cohort this percentage is even lower (20%), counterwise, our cohort was constituted mostly of patients with advanced disease at diagnosis (96%). This may explain the differences in the mutagenic profile, for instance our cohort had a higher mutation rate for *MLL2, KEAP1, NF1, PIK3CA*, STK11, and *TP53* and a lower mutation rate for STK11*, PTEN*, KRAS, HRAS, and *SOX2*. There were no mutations in genes such as *NFE2L2, FOXP1* among others. The variations in the genomic and epigenomic landscape of lung SCC according to stage of the disease has not been studied, this would be an interesting area of research in the future and would help increase our understanding of the disease. Furthermore, the sex trends visible in the North American and Korean cohorts reflect the sex distribution described for lung SCC (44% cases are men and 25% woman) (22) while our cohort displayed an almost 1:1 female to male ratio, possibly due to the limited number of patients present in our study.

**Figure 4:**
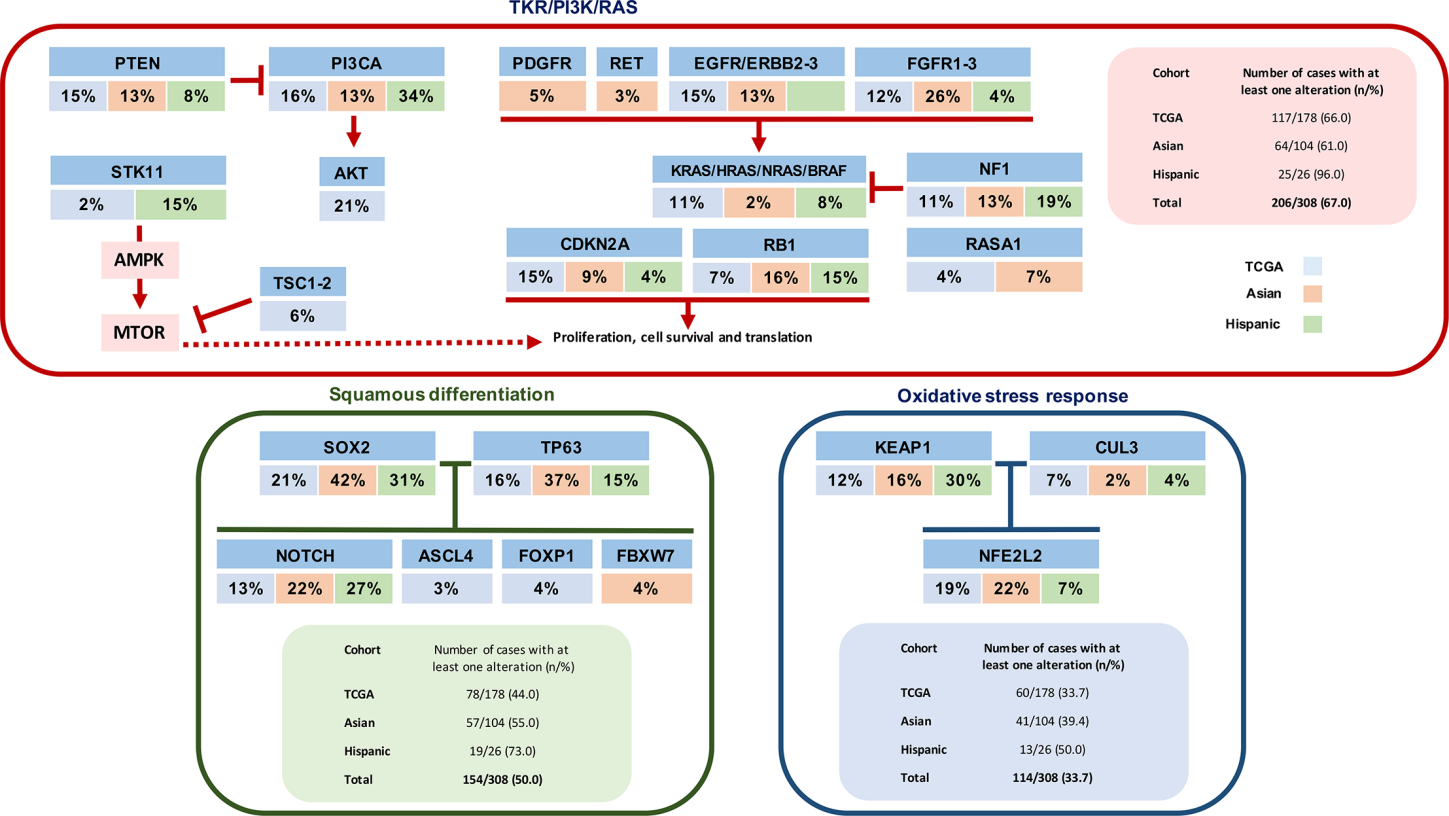
Diagram integrating three different core pathways of lung squamous cell carcinoma (considering information of substitution mutations, truncating mutations, amplifications, and deletions) from the information provided by Korean cohort (18) and by TCGA (16).

Another interesting area in lung SCC is its immunogenic profile. The PD-1 and PD-L1 pathway works as an immune inhibitory checkpoint. Tumors express these molecules and consequently evade the anti-tumoral immune response (23, 24). PD-1/PD-L1 inhibitors are pharmacologic agents that block this immune checkpoint and have shown promising activity in advanced NSCLC (25–29). Takada et al. studied the expression of PD-L1 among lung SCC patients that underwent surgical resection and found that 51.7, 35.1, 29.7, and 18.0% patients were positive for PD-L1 expression at cut off values of 1, 5, and 50% respectively. (30) There are significant differences in the prevalence of PD-L1 expression present in our data, and other reported in the literature (18, 31–34). Yet, to date there are no standardized methods (multiple monoclonal antibodies for IHC evaluation of PD-L1 expression are available) or definitions for PD-L1 positivity interpretation. This poses great difficulty on the interpretation and comparisons of the various clinical studies. Other potential causes for this variability are patient demographics, therapies and stage of the disease in the study population. (35) In our study, patients with PD-L1 expression had a slightly prolonged OS in comparison with those who had negative expression. In the literature, the prognostic value of PD-L1 expression is still controversial, multiple studies show contradictory results, again possibly due to the lack of standardization of IHC PD-L1 evaluation. (30) Another gene which is closely related with NSCLS is TP53, which is known as a tumor suppressor gene that, when defective, leads to an abnormal proliferation of cells resulting in cancer. Interestingly, in our study, we saw a positive correlation between TP53 at a lower expression of PD-L1. However, in the literature as presented by Maher et al**., TP53 was associated with an induction of PD-L1 expression, contrary to our results (36). Additionally, TP53 mutation carriers have earlier development of metastases and this, as in our study, is associated with worse prognosis. Similar conclusions were published by Carlisle et al**, where the impact of this oncogene in immunotherapy-treated patients was studied, with results also showing poor efficacy in mutation carriers (37). Furthermore, PI3KCA is an oncogene that is overexpressed in NCLS and other types of cancer, and has been related to increased cell proliferation. It was an important marker in our study, as mutations of P13KCA are associated with greater PD-L1expression. Additionally, a prolonged PFS of approximately 3 months was observed for patients who presented this association. In a meta- analysis where the co-occurrence of these two markers were analyzed, no relationship was found (38). It should be mentioned that these results may have confounding factors, given that it was performed in different histologically cohorts, both LUAD and in SCC, in a population with demographic characteristics different from those of our study. It is noteworthy to mention that this positive relationship seen in our cohort could potentially be used as a therapeutic target, where the induction of mutation in PI3KCA could increase the sensitivity to immunotherapy, allowing better responses and outcomes to treatments. **Figure 4** presents a diagram integrating three different core pathways of lung squamous cell carcinoma.

Other factors related to the immunogenic profile of SCC that may explain the efficacy of PD-1/PD-L1 inhibitors seen in this malignancy is its high mutation rate that probably causes expression of a large amount of tumor antigens. Moreover, the TCGA trial identified previously unreported loss of function mutations in the HLA-A class I major histocompatibility gene, and other genomic alterations in genes that govern cellular immunity and tumoral immune evasion including *HLA-A, HLA-B, HLA-C, B2M, MICA, MICB, ULBP1*, and *ULBP2* have also been described (16, 39). Some authors have proposed that these mutations in the *HLA-A* gene may serve as a genotypic selection marker for the use of this immunotherapy (16).

## Conclusions

Our study identified previously described mutations among Hispanic patients with SCC. Lower PDL1 expression was also found among those who had alterations in TP53 and high expression of PIK3CA a major expression of PDL-1. This further supports the notion that SCC is a highly mutated malignancy that apparently is homogeneous among diverse demographic populations. This high mutagenic and immunogenic profile renders multiple potential therapeutic targets that may impact the overall prognosis of this disease. More studies with larger subject populations are required to further characterize the genomic, epigenomic, and immunogenic of SCC, and continue to impact on the natural history of the disease.

## Data Availability Statement

The datasets presented in this article are not readily available because of the Colombian organic law of data protection that limits access to raw genetic information in an open format. Requests to access the datasets should be directed to the corresponding author, who will release it upon formal request to the Ministry of Health of Colombia following the requirements of Law 1581 of 2012, paragraph 201811601170851 of 2018.

## Ethics Statement

The studies involving human participants were reviewed and approved by Geno1.1-CLICaP Platform - Registration No. 2011/048, Clínica del Country, Bogotá, Colombia. Written informed consent for participation was not required for this study in accordance with the national legislation and the institutional requirements. 

## Author Contributions

AC, AR-P, OA, LC, CM, CR, and RR planned and coordinated the study. ZZ-B, LRo, GR, FB, CS, MBr, NZ, and LV reviewed patient records and composed the database. LRi reviewed all histopathology studies. JR, JA, PA, MBr, MBe, and TG performed DNA and RNA extraction and library preparation, NGS sequencing and PD-L1 IHC. AC, AR-P, OA, LC, CM, LP, and RR performed all statistical analysis and data interpretation. AC, AR-P, CS, and ZZ-B wrote the initial draft of the manuscript. All authors contributed to the article and approved the submitted version.

## Funding

The present study was funded by the Foundation for Clinical and Applied Cancer Research (FICMAC), Bogotá, Colombia, and the Latin American Consortium for Lung Cancer Research (CLICaP).

## Disclaimer

Preliminary results from this study were previously presented during the 18^th^ World Conference on Lung Cancer – IASLC (October 4–10, 2017, Yokohama, Japan).

## Conflict of Interest

The authors declare that the research was conducted in the absence of any commercial or financial relationships that could be construed as a potential conflict of interest.

The reviewer AA declared a past co-authorship with one of the authors CR to the handling editor.
