# Supplementary material for: Genotyping Squamous Cell Lung Carcinoma in Colombia (Geno1.1-CLICaP)
Source: Front Oncol. 2020 Dec 15;10:588932. doi: 10.3389/fonc.2020.588932 (PMC7771515; doi:10.3389/fonc.2020.588932)
Supplement: Supplementary file 1 [file Table_1.pdf]

Supplementary Tables

Supplementary Table 1. Gene list of the TruSight Tumor 170 (TST-179) panel assay including small nucleotide variants and indels (from DNA), copy number variation (from DNA) and fusions and splice variants (from RNA).

**Small nucleotide variants and indels**
AKT1 BRIP1 CREBBP FANCI FGFR2 JAK3 MSH3 PALB2 RAD51B TET2 AKT2 BTK CSF1R FANCL FGFR3 KDR MSH6 PAX3 RAD51C TMPRSS2 AKT3 CARD11 CTNNB1 FBXW7 FGFR4 KIT MTOR PAX7 RAD51D TP53 ALK CCND1 DDR2 FGF1 FLT1 KMT2A MUTYH PDGFRA RAD54L TSC1 APC CCND2 DNMT3A FGF2 FLT3 KRAS MYC PDGFRB RB1 TSC2 AR CCNE1 EGFR FGF3 FOXL2 MAP2K1 MYCL1 PIK3CA RET VHL<br>ARID1A CD79A EP300 FGF4 GEN1 MAP2K2 MYCN PIK3CB RICTOR XRCC2 ATM CD79B ERBB2 FGF5 GNA11 MCL1 MYD88 PIK3CD ROS1 ATR CDH1 ERBB3 FGF6 GNAQ MDM2 NBN PIK3CG RPS6KB1 BAP1 CDK12 ERBB4 FGF7 GNAS MDM4 NF1 PIK3R1 SLX4 BARD1 CDK4 ERCC1 FGF8 HNF1A MET NOTCH1 PMS2 SMAD4 BCL2 CDK6 ERCC2 FGF9 HRAS MLH1 NOTCH2 PPP2R2A SMARCB1 BCL6 CDKN2A ERG FGF10 IDH1 MLLT3 NOTCH3 PTCH1 SMO BRAF CEBPA ESR1 FGF14 IDH2 MPL NPM1 PTEN SRC BRCA1 CHEK1 EZH2 FGF23 INPP4B MRE11A NRAS PTPN11 STK11 BRCA2 CHEK2 FAM175A FGFR1 JAK2 MSH2 NRG1 RAD51 TERT
**Copy number variation**
AKT2 BRCA2 CHEK1 ERCC2 FGF5 FGF14 FGFR MDM4 NRG1 RAF1 ALK CCND1 CHEK2 ESR1 FGF6 FGF19 JAK2 MET PDGFRA RET AR CCND3 EGFR FGF1 FGF7 FGF23 KIT MYC PDGFRB RICTOR ATM CCN21 ERBB2 FGF2 FGF8 FGFR1 KRAS MYCL1 PIK3CA RPS6KB1 BRAF CDK4 ERBB3 FGF3 FGF9 FGFR2 LAMP1 MYCN PI3K TFRC BRCA1 CDK6 ERCC1 FGF4 FGF10 FGFR3 MDM2 NRAS PTEN
**Fusions and splice variants**
ABL1 BRAF EML4 ETV4 FGFR4 KIF5B MYC NTRK3 PIK3CA TMPRSS2 AKT3 BRCA1 ERBB2 ETV5 FLI1 KIT NOTCH1 NTRK3 PPARG ALK FRCA2 ERG EWSR1 FLT1 KMT2A NOTCH2 PAX3 RAF1 AR CDK4 ESR1 FGFR1 FLT3 MET NOTCH3 PAX7 RET

AXL	CSF1R	ETS1	FGFR2	JAK2	MLLT3	NRG1	PDGFRA	ROS1
BCL2	EGFR	ETV1	FGFR3	KDR	MSH2	NTRK1	PDGFRB	RPS6KB1
