# Supplementary figures and images for: Genotyping Squamous Cell Lung Carcinoma in Colombia (Geno1.1-CLICaP)
Source: Front Oncol. 2020 Dec 15;10:588932. doi: 10.3389/fonc.2020.588932 (PMC7771515; doi:10.3389/fonc.2020.588932)

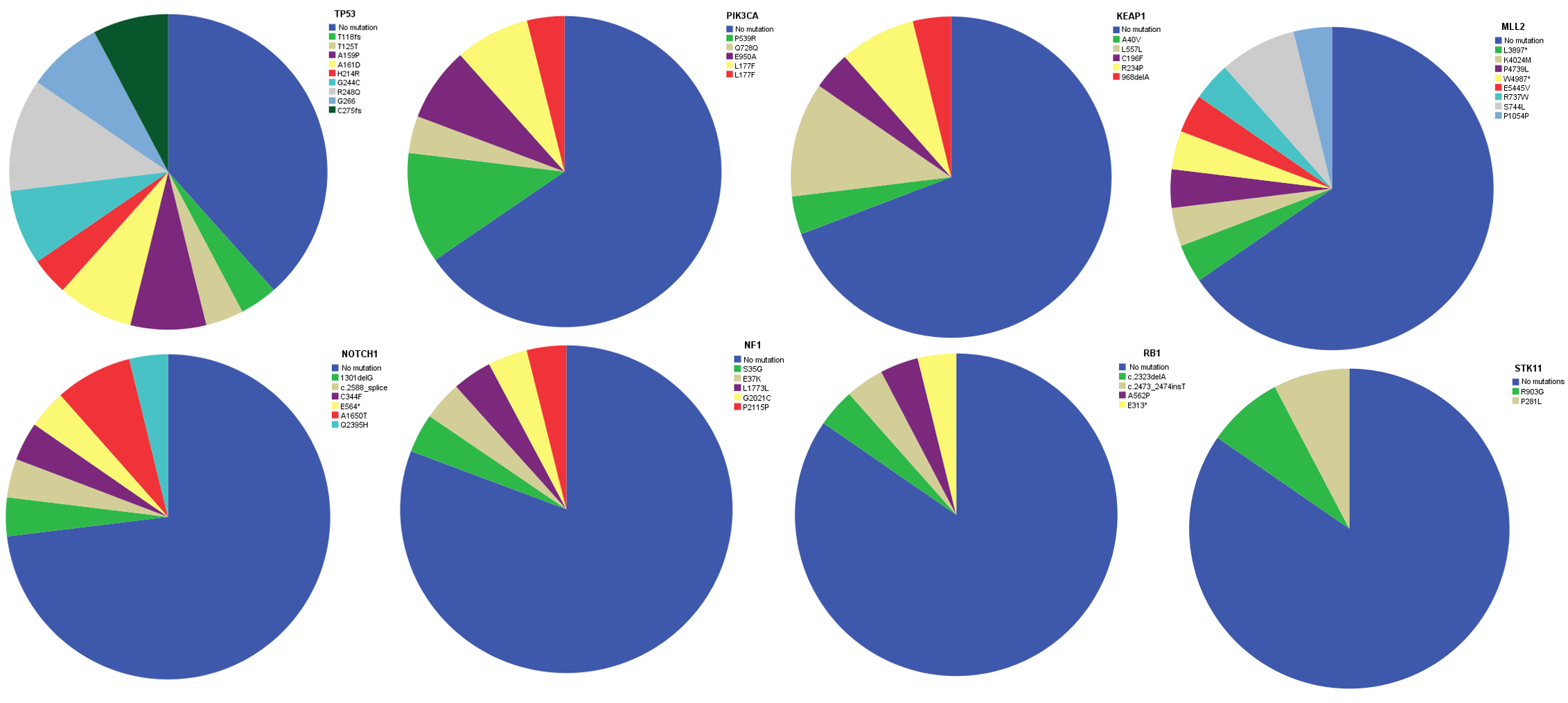

Supplement: Supplementary file 2 [file Image_1.tif]
